# Long-Term Outcomes of Radio-Frequency Catheter Ablation on Ventricular Tachycardias Due to Arrhythmogenic Right Ventricular Cardiomyopathy: A Single Center Experience

**DOI:** 10.1371/journal.pone.0169863

**Published:** 2017-01-25

**Authors:** Wei Wei, Hongtao Liao, Yumei Xue, Xianhong Fang, Jun Huang, Yang Liu, Hai Deng, Yuanhong Liang, Zili Liao, Fangzhou Liu, Weidong Lin, Xianzhang Zhan, Shulin Wu

**Affiliations:** 1 Guangdong Cardiovascular Institute, Guangzhou, P.R. China; 2 Guangdong Provincial Key Laboratory of South China Structural Heart Disease, Guangzhou, P.R. China; 3 Guangdong General Hospital, Guangdong Academy of Medical Sciences, Guangzhou, P.R. China; Scuola Superiore Sant'Anna, ITALY

## Abstract

**Aims:**

To summarize our experience of radiofrequency catheter ablation (RFCA) for recurrent drug-refractory ventricular tachycardias (VTs) due to arrhythmogenic right ventricular cardiomyopathy (ARVC) in our center over the past 11 years and its related factors.

**Methods and Results:**

We reviewed 48 adults (mean age 39.9 ± 12.9 years, range: 14 to 65) who met the present ARVC diagnostic criteria and accepted RFCA for VTs from December 2004 to April 2016. The patients received a total of 70 procedures using two ablation approaches, the endocardial approach in 52 RFCAs, and the combined epicardial and endocardial approach (the combined approach) in 18 RFCAs. Kaplan-Meier survival analysis showed that the combined approach achieved better acute procedural success (*p* = 0.003) and better long-term outcomes (*p* = 0.028) than the endocardial approach. Patients who obtained acute procedural success with non-inducibility had better long-term outcomes (*p* < 0.001). COX regression of multivariate analysis showed that procedural success was the only factor that benefited long-term outcome, irrespective of the endocardial or the combined approach (*p* = 0.001). The rate of sudden cardiac death (SCD) in patients without procedural success was significantly higher than that in patients with procedural success (*p* = 0.005). All patients without implantable cardioverter defibrillator (ICD) implantation who had successful final RFCA survived.

**Conclusions:**

The combined approach resulted in better procedural success and long-term VT-free survival compared with the endocardial approach in ARVC patients with recurrent VTs. Acute procedural success with non-inducibility was strongly related to better long-term VT-free survival and reduced SCD, irrespective of whether this was achieved by the endocardial approach or the combined approach.

## Introduction

Arrhythmogenic right ventricular cardiomyopathy (ARVC) is a genetically determined cardiomyopathy which is characterized pathologically by fibro-fatty replacement of the right ventricular (RV) myocardium[1]. Progressive and diffuse structural abnormalities can provide a substrate for reentrant ventricular tachycardias (VTs) which usually cause unstable hemodynamics. The guidelines recommend routine implantable cardioverter defibrillator (ICD) implantation in these patients, while radiofrequency catheter ablation (RFCA) is performed in patients with recurrent VTs despite anti-arrhythmic drugs[2]. With respect to mapping and ablation techniques, there are two approaches used to reach the myocardium, the endocardial approach and the epicardial approach. However, the long-term effects of RFCA in these patients are unclear. The main purpose of this retrospective study was to determine the long-term outcome of RFCA in patients with VTs due to ARVC.

## Methods

This retrospective study was approved by the Research Ethics Committee on Human Research at Guangdong General Hospital, Guangdong Academy of Medical Sciences, China before the data were summarized. The patients’ records/information were anonymized and de-identified prior to analysis.

### Study population

According to the modified criteria for diagnosis of ARVC proposed by an international working group in 2010[3], we retrospectively studied 48 adults (≥ 14 years of age) who met the diagnostic criteria for ARVC and who underwent an electrophysiological study (EPS) and RFCA due to drug-refractory VTs in our hospital from December 2004 to January 2015. All patients met at least two major, or one major plus two minor, or four minor diagnostic criteria from the following different categories: i) Global or regional dysfunction and structural alterations; ii) Tissue characterization of ventricular wall; iii) Repolarization abnormalities; iv) Depolarization/conduction abnormalities; v) Arrhythmias and vi) Family history. Ablations were performed following failed medical treatment with at least two anti-arrhythmic drugs, respectively, including one type-III anti-arrhythmic drug. Failed therapy was defined as recurrent persistent VTs / syncope / pre-syncope or < 50% decrease in ventricular arrhythmia burden demonstrated by 24-h Holter monitoring.

### Pre-procedural preparation

All patients underwent echocardiography (UCG) and 12-lead surface electrocardiography (ECG) before RFCA. Their medical histories and family histories were recorded. Twenty-two patients underwent contrast-enhanced cardiac magnetic resonance (CMR) imaging using a clinical 1.5-T scanner, the CMR data consisting of right ventricular end-diastolic volume (RVEDV) and right ventricular ejection fraction (RVEF) were available for 15 patients. Right ventricular end-diastolic volume index (RVEDVI) was assessed using the following calculation: RVEDVI = RVEDV / body surface area. Body surface area (m^2^) = 0.0061×height (cm)+0.0128×weight (kg)-0.1529. All patients signed informed consent forms before RFCA. All patients were recommended for ICD implantation on diagnosis unless they refused. Anti-arrhythmic drugs were stopped at least 5 half-lives preoperatively.

### Electrophysiological study, mapping and ablation techniques

A quadripolar-electrode catheter (with inter-electrode spacing of 10 mm) was placed in the RV via the right femoral vein. A decapolar electrode catheter (with an inter-electrode spacing of 2–5–2 mm) was placed in the coronary sinus (CS) via the left subclavian vein. A programmed electrophysiological study (PES) was carried out. The standard protocol for induction of VTs included programmed stimulation with up to three ventricular extra-stimuli delivered from at least two RV sites, starting at S1S2 = 500 ms / 350 ms—10 ms. Surface ECGs and bipolar/unipolar intra-cardiac electrograms were monitored on a LabSystem Pro (Bard Electrophysiology, Lowell, MA, USA). Signals were sampled at 1 kHz and filtered at 0.1 to 50 Hz for surface ECGs and 30 to 500 Hz for intra-cardiac signals, and displayed at an amplification of 0.1 mV/cm. Three-dimensional electro-anatomical mapping systems (Carto XP / Carto 3, Biosense Webster, Diamond Bar, CA, US and Ensite NAVX, St. Jude Medical, Minneapolis, MN, USA) were used.

**Fig 1:**
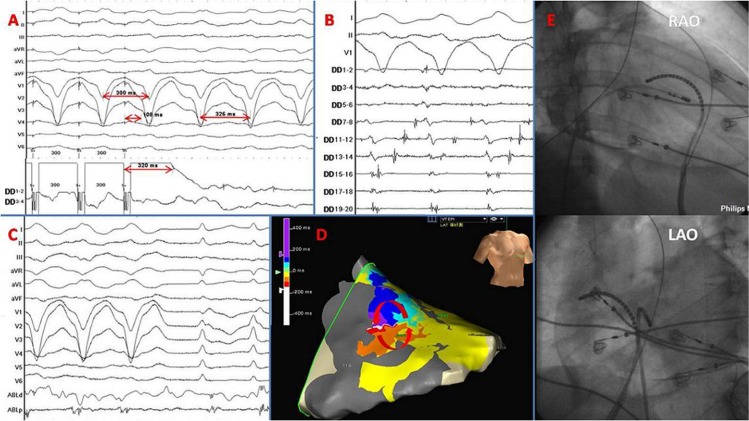
This series of pictures show the electrophysiological characteristics in the RV epicardium of an ARVC patient. (A) A concealed entrainment within the isthmus of the reentrant circuit. The post-pacing interval (PPI) was 320 ms, almost equal to the VT cycle length (326 ms). The interval between the stimulation signal to onset of QRS complex (S-QRS) was 108 ms (108/320 = 0.34), indicating that the location was within the center of the isthmus. (B) The sequential activation of fractionated potentials. (C) VT stopped during ablation. (D) The three-dimensional activation map (Ensite Navx Velocity) of this patient’s RV epicardium which shows a reentry in the RV free wall. (E) Fluorescence of the mapping catheters. DD: St Jude Medical DuoDeca catheter.

**Fig 2:**
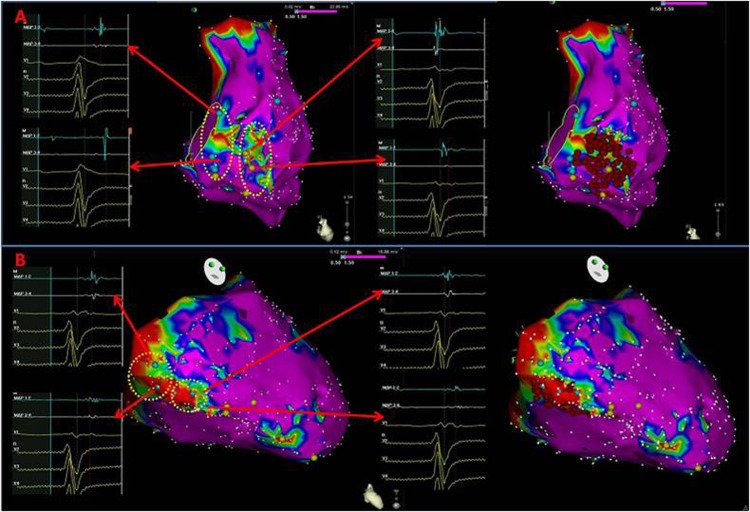
The substrate maps of the RV endocardium and epicardium of an ARVC patient (CARTO3 system). (A) The fractionated potentials are marked as blue dots within a low voltage zone and ablation energy was delivered within the low voltage zone with fractionated potentials in the RV endocardium. (B) This is the substrate map of the RV epicardium and ablation marks within the low voltage zone with fractionated potentials of the same patient.

Conventional mapping and ablation techniques included pace mapping, activation mapping, voltage mapping and entrainment (Fig 1). Activation mapping and entrainment were used for sustained VTs. Pace mapping and voltage mapping during sinus rhythm (SR) were applied for non-sustained VTs. The voltage criterion for normal myocardium was > 1.5 mV. The voltage criterion for dense scarring was < 0.5 mV[4]. Between these two values was defined as transitional scarring. Substrate modification targeting late potentials and fractionated potentials was applied (Fig 2), which were designated by Haïssaguerre and colleagues as local abnormal ventricular activities (LAVAs)[5]. LAVAs were defined as sharp high-frequency ventricular potentials, possibly of low amplitude, distinct from the far-field ventricular electrogram occurring anytime during or after the far-field ventricular electrogram in SR or before the far-field ventricular electrogram during VT that sometimes displayed fractionation or double or multiple components separated by very-low-amplitude signals or an iso-electric interval and were poorly coupled to the rest of the myocardium. Clinical VTs were defined as the match of induced VTs to documented VTs in ≥ 11 leads.

Epicardial mapping has been used in our center for ARVC patients since 2011. The epicardial approach was used if any one of the following four items existed: i) Failed previous endocardial procedures. ii) The epicardial origin was suspected based on features of VT morphologies including Q wave in inferior leads for VTs originating from the RV inferior wall, and Q wave in lead I or QS type of lead V2 for VTs originating from the RV anterior wall. iii) The endocardial approach failed to obtain the best paced map or concealed entrainments of sustained VTs. iv) The endocardial substrate did not show abnormal potentials. The epicardial approach was achieved through an anterior subxyphoid puncture as described by Sosa et al[6], guided by a 45° left anterior oblique (LAO) fluoroscopic projection. A 10F sheath or an Agilis sheath was placed in the pericardium as soon as the pericardial cavity was accessed.

The definition of acute procedural success was no induction of clinical VTs and any sustained VTs (lasting for up to 30 s) by PES with intravenous isoprenaline (1–3 μg / min to increase 20%– 25% of baseline heart rate) and atropine (1 mg). Otherwise, the procedure was considered unsuccessful, even if some of the documented VTs disappeared after RFCA.

A pigtail catheter was inserted into the pericardial cavity for drainage of effusion for at least 24 h post-procedure. A 5 mg bolus of dexamethasone was injected into the pericardial cavity for prevention of adhesions.

### Follow-up

We prescribed 1 to 2 anti-arrhythmic drugs such as metoprolol, amiodarone and sotalol for all patients within 3 to 6 months after RFCA, unless they experienced recurrence and redo procedures during this period. All patients were followed-up regularly at 1 month, 3 months, 6 months, 1 year post-procedure, and then every year. Twelve-lead ECG, trans-thoracic echocardiography (TTE) and 24-h ambulatory ECG monitoring (Holter) were reviewed during follow-up. The criteria for VT recurrence were as follows: i) Persistent VTs (> 30 s) documented by Holter; ii) Related symptoms of VTs/VF of any morphology (even different from the previous morphology) documented by emergent ECG; iii) Syncope or sudden cardiac death (SCD). On recurrence, a redo procedure and ICD implantation were recommended and a more frequent follow-up was carried out.

### Statistical analysis

Continuous variables are presented as mean ± standard deviation. Repeat procedures performed on the same patient were assumed to be independent. Comparisons of cumulative VT-free survival were analyzed by the log-rank test using data from the Kaplan-Meier survival analysis. Comparisons of the success of different approaches were analyzed by Fisher’s exact test. Multivariate analysis was carried out by COX regression analysis. The factors associated with VT-free survival were screened by univariate analysis and those with a value of *p* < 0.20 were further analyzed by multivariate analysis. Two-tailed *p* < 0.05 was considered significant. SPSS 19.0 statistical software was used for statistical analysis.

## Results

### Patient characteristics

The percentage of ARVC among all patients undergoing ventricular arrhythmia ablations during the same period in our center was 48 / 2834 = 1.69%. Thirty-three men and 15 women, mean age 39.9 ± 12.9 years (range: 14 to 65), were undergoing their first procedure. The mean right ventricular ejection fraction (RVEF) was 31.2 ± 11.5% and the left ventricular ejection fraction (LVEF) was 51.4 ± 8.0%. The RVEDVI was 159.1 ± 44.7 ml/m^2^. The mean recorded history of tachycardia was 4.1 ± 6.6 years before admission. Twelve patients (25.0%) had severe symptoms during VTs including pre-syncope, syncope, and even VF. Six patients experienced a failed endocardial RFCA in other hospitals before being referred to us. Five patients had suffered symptomatic right heart failure of NYHA grade 2 to 3 before RFCA. Five patients accepted ICD implantation before their first RFCA, while six patients accepted ICD implantation after their first RFCA due to recurrence. The remaining 37 patients refused ICD implantation due to personal reasons. The general status of these patients is shown in Table 1. In total, 30 (62.5%) patients underwent the endocardial approach, while 18 (37.5%, including one patient who had undergone a combined procedure in another center) patients received the combined approach from April 2011. In 22 RFCAs, thermo-control catheters were used and irrigated catheters were used in 48 RFCAs (S1 and S2 Tables). After all 70 procedures, we prescribed amiodarone for 35 procedures, metoprolol for 21, amiodarone and metoprolol for nine, and sotalol for five. Prescriptions of anti-arrhythmic drugs were altered during observation. For 44 surviving patients (4 died), only 31 (31 / 44 = 70.5%) insisted on taking anti-arrhythmic drugs chronically such as metoprolol, sotalol, amiodarone and dronedarone. The other 13 patients discontinued anti-arrhythmic drugs due to personal reasons.

**Table 1:** General demographic data and clinical status.

Items	Values (mean± SD)
**Age (years)**	39.9 ± 12.9
**Gender**	M 33, W 15
**Manifest history (years)**	4.1 ± 6.6
**Symptomatic right heart failure**	5
**RVEDVI (ml/m**^**2**^**) by MRI**	159.1 ± 44.7
**RVEF (%) by MRI**	31.2 ± 11.5
**LVEF (%) by MRI**	51.4 ± 8.0
**ICD implantation**	11
**Average follow-up time (month)**	71.4 ± 45.7

### Acute and long-term outcomes

In total, 48 patients underwent 70 procedures (1 to 4 procedures per patient), including 18 combined procedures and 52 endocardial procedures. We induced a total of 77 VTs at the following locations: i) 25 on the free wall of the right ventricular inflow tract (RVIT-FW); ii) 20 on the inferior wall of the right ventricular inflow tract (RVIT-IW); iii) 23 on the right ventricular outflow tract (RVOT); iv) 6 on the right ventricular apex (RVA); v) 3 on the left ventricular apex (LVA). Forty-five VTs (i+ii: 45 / 77 = 58.4%) were located on the peri-tricuspid valve. Thirty-nine (39 / 48 = 81.3%) patients achieved acute procedural success following their final or only RFCA, while nine patients did not (9 / 48 = 18.7%).

The average follow-up duration was 71.4 ± 45.7 months (median 79.5 months). In total, 27 (27 / 48 = 56.3%) patients have remained asymptomatic since their last procedure without documented VT recurrence (recorded either by repeated 24 h Holter or ICD). To date, only 18 (18 / 48 = 37.5%) of these patients have enjoyed VT-free survival after a single procedure. The mean VT-free survival time in these 27 patients was 49.3 ± 36.9 months (range: 3 to 126 months). The other 21 (21 / 48 = 43.7%) patients suffered recurrences after their last procedure (1 to 3 procedures per patient). The mean time to VT recurrence was 13.4 ± 27.2 months for all procedures (S2 Table).

### Results of Kaplan-Meier survival analysis and COX regression analysis

**Fig 3:**
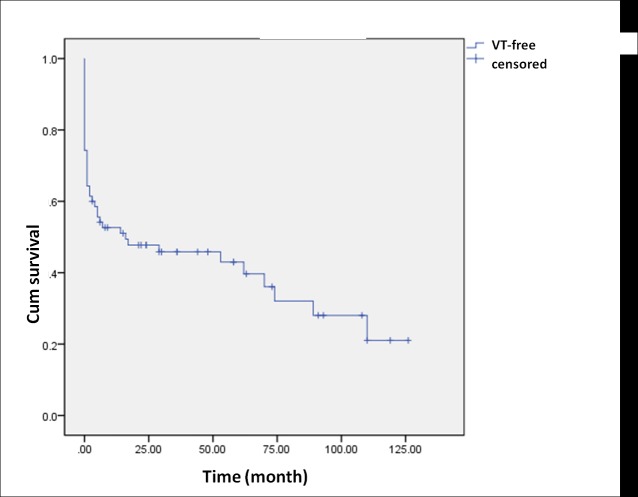
The overall cumulative VT-free survival curve of all 70 procedures.

**Fig 4:**
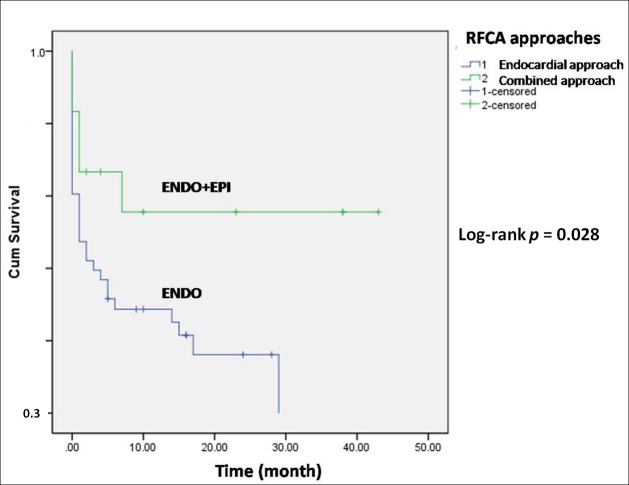
The cumulative VT-free survival curves of different RFCA approaches (the endocardial approach and the combined approach). The latter achieved better results than the former (Log-rank *p* = 0.028).

**Fig 5:**
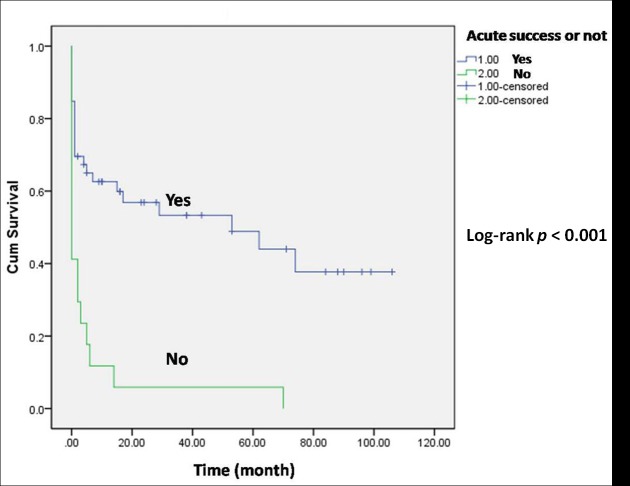
The cumulative VT-free survival of different acute results. The procedures leading to acute procedural success achieved significantly better VT-free survival than those without acute procedural success (Log-rank *p* < 0.001).

Fig 3 shows the overall cumulative VT-free survival after all 70 procedures. A sharp decrease in VT-free survival was observed in the early stage. The combined approach resulted in better cumulative VT-free survival than the endocardial approach (Log-rank *p* = 0.028, Fig 4). There was a significant difference in the acute procedural success rate between the combined approach and the endocardial approach (Fisher exact test *p* = 0.003). The procedures which resulted in acute procedural success showed significantly better VT-free survival (Log-rank *p* < 0.001, Fig 5).

COX regression analysis was carried out on the data from all 43 patients. Univariate analysis showed that ablation approach (HR = 0.389, 95% CI = 0.152–0.998, *p* = 0.049), acute procedural success (HR = 0.116, 95% CI = 0.224–0.432, *p* < 0.001), number of RFCAs (1 RFCA vs. more, HR = 1.663, 95% CI = 0.848–3.262, *p* = 0.139), catheter type (thermo-controlled catheter vs. irrigated catheter, HR = 1.624, 95% CI = 0.880–3.000, *p* = 0.121) and number of VTs (HR = 0.605, 95% CI = 0.302–1.038. *p* = 0.065) were likely related to VT-free survival. Other factors including gender, age (< 40 y or > 40 y), RVEDI, RVEF and LVEF were unlikely to be related to VT-free survival (*p* > 0.20 in all). These five factors (acute procedural success, ablation approach, catheter type, number of RFCAs and number of VTs), which were associated with VT-free survival (*p* < 0.2) were further analyzed by multivariate analysis. The combined approach was not associated with better VT-free survival than the endocardial approach (HR = 0.641, 95% CI = 0.224–1.839, *p* = 0.408). These results were different to those in the univariate analysis; however, acute procedural success was associated with better VT-free survival (HR = 0.286, 95% CI = 0.132–0.618, *p* = 0.001) (Table 2). We then performed a COX regression analysis of data from patients who had only undergone the endocardial approach. Univariate analysis showed that acute procedural success (HR = 0.250, 95% CI = 0.123–0.507, *p* < 0.001) and the number of RFCAs (HR = 0.609, 95% CI = 0.300–1.238, *p* = 0.170) were likely related to VT-free survival. Other factors including catheter type, number of VTs, gender, age, RVEDI, RVEF and LVEF were unlikely to be related to VT-free survival (*p* > 0.20 in all). Further multivariate analysis of acute procedural success and the number of RFCAs showed that only the former was significantly related to VT-free survival (HR = 0.264, 95% CI = 0.122–0.569, *p* = 0.001) (Table 3).

**Table 2:** Multivariate analysis of VT-free survival in all patients by COX regression analysis.

Variables	Univariate analysis		Multivariate analysis	
	HR (95%CI)	*p* value	HR (95%CI)	*p* value
**Approaches**	0.389 (0.152–0.998)	0.049*	0.641(0.224–1.839)	0.408
**(Combined vs. Endo)**				
**Acute procedural success**	0.116 (0.224–0.432)	< 0.001*	0.286(0.132–0.618)	0.001*
**RFCA times (1**^**st**^ **RFCA vs. more)**	1.663 (0.848–3.262)	0.139	1.368(0.679–2.759)	0.381
**Catheter types**	1.624 (0.880–3.000)	0.121	1.068(0.541–2.106)	0.85
**(thermo-control vs. irrigated)**				
**VT numbers**	0.560 (0.302–1.038)	0.065	0.904(0.454–1.802)	0.774
**(≤2 VTs or > 2 VTs)**				
**Gender**	1.327 (0.689–2.556)	0.397		
**Age (< 40y or > 40 y)**	1.075 (0.589–1.961)	0.813		
**RVEDVI**	1.003 (0.985–1.022)	0.728		
**RVEF**	0.968 (0.997–1.045)	0.411		
**LVEF**	1.005 (0.895–1.129)	0.931		

* represents a statistically significant *p* value.

**Table 3:** Multivariate analysis of VT-free survival in the endocardial approach group by COX regression analysis.

Variables	Univariate analysis		Multivariate analysis	
	HR (95%CI)	*p* value	HR (95%CI)	*p* value
**Acute procedural success**	0.250 (0.123–0.507)	< 0.001*	0.264(0.122–0.569)	0.001*
**RFCA times (1**^**st**^ **RFCA vs. more)**	0.609 (0.300–1.238)	0.170	0.767(0.365–1.613)	0.485
**Catheter types**	0.791 (0.413–1.517)	0.481		
**(thermo-control vs. irrigated)**				
**VT numbers**	1.584 (0.819–3.066)	0.172	1.001(0.485–2.069)	0.997
**(≤2 VTs or > 2 VTs)**				
**Gender**	0.792 (0.403–1.553)	0.497		
**Age (< 40y or > 40 y)**	1.074 (0.566–2.036)	0.828		
**RVEDVI**	0.979 (0.933–1.026)	0.252		
**RVEF**	1.135 (0.881–1.464)	0.327		
**LVEF**	2.356 (0.389–14.266)	0.351		

* represents a statistically significant *p* value.

### SCD and progression of heart failure

Three patients died (3 / 48 = 6.3%) of SCD during follow-up. They died 1 month, 4 months and 20 months after their last unsuccessful RFCA, respectively. The VT locations in these patients were in the RVOT, RVIT+RVOT, and RVOT, respectively. All procedures were via the endocardial approach (3 / 30 = 10.0% of all endocardial patients). Thus, the SCD rate due to malignant arrhythmias in patients without acute procedural success following the last RFCA was 33.3% (3 / 9), while the SCD rate was 0% (0 / 39) in patients with acute procedural success following the last RFCA (*p* = 0.005, by Fisher’s exact test). None of these three patients had accepted ICD implantation, while 11 patients who accepted ICD implantation (nine with acute procedural success following the last RFCA, two without acute procedural success) either before or after RFCA are still alive. Among the patients with ICD implantation, five have enjoyed VT-free survival since the last RFCA, while recurrence was observed in the other six patients. The difference in SCD rates between the patients with and without ICD was not statistically significant (*p* = 1.0, by Fisher’s exact test). In total, 30 patients without ICD implantation achieved acute procedural success following their last RFCA and all survived (100%), whether they experienced VT recurrence (7 / 30 = 23.3%) or not (23 / 30 = 76.7%). Eight patients without ICD implantation failed their last RFCA and three of them experienced SCD (3 / 8 = 37.5%). The difference between these two sub-groups was statistically significant (*p* = 0.007, by Fisher’s exact test).

In the five patients with symptomatic right heart failure pre-procedure, ablation did not improve their condition, and three of these patients suffered recurrences. Although heart function in two patients progressed from NYHA grade 3 pre-procedure to NYHA grade 4 post-procedure, no VT recurrences were documented, and heart transplantation is recommended for these patients. One of these patients received a heart transplant in December 2014, but died of graft versus host disease in February 2015 (S2 Table).

### Complications

None of the patients experienced obvious pericardial bleeding (> 200 ml drainage per day) due to epicardial ablation, life-threatening major bleeding (including cerebral hemorrhage, gastrointestinal bleeding, cardiac tamponade, etc.), cardiac rupture due to catheter manipulation or myocardial infarction due to impairment of the coronary arteries.

## Discussion

Ventricular arrhythmias in the setting of ARVC are compatible with reentry[7]. The areas of scarred myocardium in ARVC are manifested by low-voltage, fragmented intra-cardiac potentials with prolonged duration[8]. The diseased ventricular tissue activates more slowly than normal tissue, which generates Epsilon waves on pre-cordial leads of surface ECG and LAVAs on electrogram. The present guidelines recommend ICD implantation for primary and secondary prevention of SCD in ARVC patients. ARVC is a progressive disease with individual differences. This helps to explain why some of our patients suffered recurrences after ablation and worsening heart function even with appropriate medications, while others remained stable with respect to both heart rhythm and heart function. According to previous reports, the long-term VT-free survival rates following ablation in such patients varied greatly, from 25% to 89% in approximately 2 years[9–13].

### RFCA strategies in ARVC patients and procedure endpoints

In addition to the help of 3D mapping systems, the conventional mapping strategy was performed via the endocardial approach, which was inadequate to determine the complex mechanisms of reentry. Previous studies have reported that the epicardium plays a more important role than the endocardium in VTs due to ARVC[9,14]. In our study, we have carried out the combined approach for ARVC since April 2011 and the overall VT-free survival has improved as shown by the results of univariate analysis.

According to the results of survival analysis and Fisher’s exact test, acute procedural success was significantly related to VT-free survival and reduced SCD. COX regression analysis further demonstrated that acute procedural success benefited VT-free survival, irrespective if patients only underwent the endocardial approach. These findings were different to those from a previous study by Dalal et al[15] which only included the endocardial approach. According to our results, acute procedural success (non-inducibility) of VT ablation in ARVC should be the desired endpoint, irrespective of how this is achieved. According to the results of multivariate COX regression analysis, acute procedural success was the only protective factor for VT-free survival, while the combined approach did not achieve similar results to those of univariate analysis. We consider that this was mainly due to the fact that the combined approach achieved a higher acute procedural success rate than the endocardial approach. As a strong predictor of long-term VT-free survival, the high acute procedural success rate in the combined approach group weakened the predictive value of the approach in multivariate analysis. Another reason may be that the RV wall is thin for RFCA power infiltration from the endocardium to the epicardium. The limited sample size in this group also affected the results. However, we still consider that the combined approach was better for both acute procedural success and long-term VT-free survival than the endocardial approach.

### Mapping maneuvers

As epicardial fatty pads have no electrophysiological activities and can amplify low-voltage areas, the voltage map of the epicardium is less accurate than that of the endocardium. Fortunately, LAVAs do not appear on fatty pads, thus are important for discriminating arrhythmogenic areas from fatty pads and normal myocardium in the epicardium[16]. Our procedures mainly focused on LAVAs within scar and border zones (between low-voltage zones and normal myocardium). Antonio Berruezo and colleagues reported a scar de-channeling technique by substrate mapping in ARVC cases[17]. They marked the conducting channels according to the timing of LAVAs. They reported that the late potential channels acted as the VT substrate in ARVC more frequently than the voltage channels and therefore should be considered for substrate-guided ablation[18]. In our experience, substrate is so complex with many crossed channels that the whole communicating pathway is difficult to display. As all the channels with concealed entrainments are potential sources of VTs, the ablation strategies for some of our cases included diffused ablation of LAVAs, especially for non-inducible and unsustained VTs. Haïssaguerre et al reported that complete elimination of LAVA could achieve better outcomes than incomplete elimination[5]. However, in our cases with diffused LAVAs, we could only reduce part of them, and not completely eliminate them.

### Considerations regarding the application of interventions

According to present guidelines for ARVC[3], ICD implantation is the first-line treatment for such patients with VTs. In the present study, most of our patients refused ICD implantation either initially or later due to personal reasons, thus we obtained data on ARVC patients who only received ablation interventions. We found that 30 patients without ICD implantation who had achieved acute procedural success following their last RFCA survived, irrespective of VT recurrence. On the other hand, three patients who suffered SCD all failed their last RFCA and did not undergo ICD implantation. Thus, we did not observe additional survival benefits due to ICD implantation in our study in the setting of successful ablation. These results raised the following questions: Is ICD implantation necessary for all ARVC patients with VT? Can ICD implantation be optional if successful ablations are achieved in experienced centers? Can patients who fail RFCA accept redo procedures before they decide to accept ICD implantation? If a patient’s VT can be controlled by RFCA then ICD implantation would be unnecessary. To answer these questions, further studies are needed.

Due to the survival of five patients with symptomatic right heart failure, we consider that RFCA can only treat VTs, but not improve heart failure, which is another main risk factor for death. Heart transplantation is the last treatment option for these patients.

### Other interfering factors in RFCA

Our results showed that the number of VTs was not related to acute procedural success. However, it is thought that the greater the number of VTs, the more difficult the condition is to manage. Our explanation for this is that the arrhythmogenic substrate of ARVC is decisive in ablation difficulties, rather than the number of VTs. The complex substrate of ARVC also causes different VT outlets. Our results showed that VT locations, which we consider to be related to catheter stability, did not significantly influence acute procedural success. RVEDVI was not associated with VT-free survival in our study which was in accordance with a previous study[19].

## Limitations

This study has several limitations. First, most patients refused ICD implantation as the first-line treatment according to present guidelines due to personal preferences and economic status. Therefore, we could not obtain continuous VT/PVC burdens in these patients during follow-up, which interfered with the quality of the data. Second, this was a single-center study with a limited sample size. Third, being a retrospective study, the mapping and ablation techniques were not uniform and could have interfered with the outcomes. Fourth, we prescribed anti-arrhythmic drugs to the patients after RFCA and we did not include a control group. Thus, further studies are needed.

## Conclusions

The combined approach resulted in better procedural success and long-term VT-free survival compared with the endocardial approach in ARVC patients with recurrent VTs. Acute procedural success with non-inducibility was strongly related to better long-term VT-free survival and reduced SCD, irrespective of whether this was achieved by the endocardial approach or the combined approach.

## Supporting Information

S1 Table General demographic data and clinical status. (DOCX)

S2 Table Procedural information and follow-up results. (DOCX)
