# Supplementary material for: Long-Term Outcomes of Radio-Frequency Catheter Ablation on Ventricular Tachycardias Due to Arrhythmogenic Right Ventricular Cardiomyopathy: A Single Center Experience
Source: PLoS One. 2017 Jan 25;12(1):e0169863. doi: 10.1371/journal.pone.0169863 (PMC5266247; doi:10.1371/journal.pone.0169863)
Supplement: S1 Table — (DOCX) [file pone.0169863.s001.docx]

**S1 Table General demographic data and clinical status**

| **Patient** | **Age (Y)** | **Gender (M/W)** | **Prior manifest history** | **Fulfilled diagnostic criteria*** | **Symptomatic**  **RV failure** | **RVEDVI by MRI** | **RVEF (%) by MRI** | **ICD** |
| --- | --- | --- | --- | --- | --- | --- | --- | --- |
| **1** | 31 | M | 4 years | 3 majors | None | 132.0 | 38.4 | No |
| **2** | 27 | W | 2 years | 2 majors, 1 minor | None | - | - | Yes, pre-RFCA |
| **3** | 50 | M | 3 months | 3 majors | None | 97.0 | 42.6 | Yes, pre-RFCA |
| **4** | 28 | M | 5 years | 3 majors | None | 93.0 | 45.0 | No |
| **5** | 38 | M | 1 year | 3 majors | None | 104.0 | 44.0 | No |
| **6** | 58 | M | 11 years | 3 majors | None | 144.0 | 29.0 | No |
| **7** | 52 | M | 2.5 months | 4 majors | None | 146.3 | 44.2 | No |
| **8** | 58 | M | 30 years | 5 majors | None | - | - | No |
| **9** | 24 | M | 2.5 years | 5 majors | None | 177.7 | 13.6 | No |
| **10** | 20 | M | 4.5 months | 5 majors | None | 245.2 | 28.0 | No |
| **11** | 37 | W | 3 days | 3 majors | None | 191.3 | 32.0 | No |
| **12** | 43 | M | 2 months | 1 major, 2 minors | None | 145.5 | 39.1 | No |
| **13** | 42 | M | 0.5 month | 4 majors, 2 minors | None | 177.7 | 37.0 | No |
| **14** | 21 | M | 1 year | 3 majors, 1 minor | None | 148.0 | 18.2 | No |
| **15** | 33 | M | 5 years | 4 majors | None | 263.7 | 15.8 | No |
| **16** | 24 | M | 3.5 years | 2 majors, 2 minors | Yes, NYHA 2 | 189.6 | 13.2 | After 1^st^ RFCA |
| **17** | 45 | W | 6 months | 2 majors | Yes, NYHA 2 | - | - | Pre-RFCA |
| **18** | 27 | M | 2 months | 2 majors | None | 116.5 | 40.1 | No |
| **19** | 49 | W | 5 years | 3 majors | None | 136.3 | 37.7 | No |
| **20** | 33 | M | 2.5 months | 3 majors | None | - | - | No |
| **21** | 41 | M | 2 years | 3 majors, 1 minor | None | - | - | No |
| **22** | 52 | W | 17 years | 4 majors, 1 minor | None | - | - | No |
| **23** | 44 | M | 1 year | 2 majors, 1 minor | None | - | - | Pre-RFCA |
| **24** | 47 | W | 4 months | 2 majors | None | - | - | No |
| **25** | 37 | M | 2.5 years | 3 majors, 2 minors | None | - | - | No |
| **26** | 63 | W | 20 years | 4 majors, 1 minor | Yes, NYHA 2 | - | - | Pre-RFCA |
| **27** | 62 | M | 1 year | 4 majors, 1 minor | None | - | - | After 1^st^ RFCA |
| **28** | 21 | M | 2 years | 3 majors | None | 135.4 | 37.0 | No |
| **29** | 29 | W | 4 years | 2 majors, 2 minors | None | - | - | No |
| **30** | 59 | M | 2 years | 3 majors, 1 minor | None | - | - | After 1^st^ RFCA |
| **31** | 41 | W | 10 years | 3 majors | Yes, NYHA 3 | - | - | After 1^st^ RFCA |
| **32** | 48 | W | 25 years | 2 majors, 2 minors | None | - | - | No |
| **33** | 50 | W | 12 years | 3 majors, 1 minor | None | - | - | After 1^st^ RFCA |
| **34** | 45 | M | 3 years | 3 majors | None | - | - | No |
| **35** | 37 | M | 6 years | 4 majors | None | - | - | No |
| **36** | 44 | M | 1 year | 3 majors | None | - | - | No |
| **37** | 62 | W | 3.5 months | 3 majors | None | - | - | No |
| **38** | 16 | M | 10 months | 2 majors | None | - | - | No |
| **39** | 65 | M | 3.5 years | 3 majors, 1 minor | None | - | - | No |
| **40** | 31 | M | 4 months | 2 majors, 1 minor | None | - | - | No |
| **41** | 34 | W | 1 year | 3 majors, 1 minor | None | - | - | After 1^st^ RFCA |
| **42** | 35 | M | 0.75 month | 2 majors, 1 minor | None | - | - | No |
| **43** | 24 | W | 5 years | 2 majors | Yes, NYHA 3 | 212.4 | 18.7 | No |
| **44** | 27 | M | 2 months | 2 majors+1 minor | None | 149.6 | 36.6 | No |
| **45** | 48 | M | 5 days | 4 majors | None | - | - | No |
| **46** | 47 | M | 2 years | 3 majors | None | - | - | No |
| **47** | 49 | M | 1.5 years | 4 majors | None | - | - | No |
| **48** | 25 | M | 2 months | 1 major+2minors | None | - | - | No |

**RFCA: radiofrequency catheter ablation; RV: right ventricle; RV-EDV: right ventricular end-diastolic volume; BSA: body surface area.**

***Diagnostic criteria of ARVC: include major and minor ones:**

1. **Global or regional dysfunction and structural alterations**

**a. 2D echo; b. MRI; c. RV angiography.**

1. **Tissue characterization of wall**
2. **Repolarization abnormalities: Surface 12-lead ECG**
3. **Depolarization/conduction abnormalities**

**a. Epsilon wave; b. Late potentials**

1. **Arrhythmias: VTs of left bundle-branch morphology with superior/inferior axis; 24h Holter.**
2. **Family history**
