# Supplementary material for: Long-Term Outcomes of Radio-Frequency Catheter Ablation on Ventricular Tachycardias Due to Arrhythmogenic Right Ventricular Cardiomyopathy: A Single Center Experience
Source: PLoS One. 2017 Jan 25;12(1):e0169863. doi: 10.1371/journal.pone.0169863 (PMC5266247; doi:10.1371/journal.pone.0169863)
Supplement: S2 Table — (DOCX) [file pone.0169863.s002.docx]

**Data 2 Procedural information and follow-up results**

| **Pts** | **RFCA No.** | **Endo/Endo+Epi approach** | **Catheter**  **type** | **Acute success** | **Follow-up months** | **Recurrence** | **Recurrent time(months)** | **Living status** |
| --- | --- | --- | --- | --- | --- | --- | --- | --- |
| **1** | 2 | Endo | I | No | 4 | Yes | 0 | Alive |
|  |  | Endo+Epi | I | Yes | 3 | No | - |  |
| **2** | 1 | Endo+Epi | I | Yes | 8 | No | - | Alive |
| **3** | 1 | Endo+Epi | I | Yes | 9 | No | - | Alive |
| **4** | 1 | Endo+Epi | I | Yes | 15 | No | - | Alive |
| **5** | 1 | Endo+Epi | I | Yes | 21 | No | - | Alive |
| **6** | 1 | Endo+Epi | I | Yes | 22 | No | - | Alive |
| **7** | 1 | Endo+Epi | I | Yes | 24 | No | - | Alive |
| **8** | 2 | Endo+Epi  Endo | I  I | Yes,  Yes | 24  24 | Yes,  No | 0,  - | Alive |
| **9** | 1 | Endo+Epi | I | Yes | 27 | Yes | 1 | Alive |
| **10** | 1 | Endo | I | Yes | 29 | No | - | Alive |
| **11** | 1 | Endo+Epi | I | Yes | 30 | No | - | Alive |
| **12** | 1 | Endo | I | Yes | 30 | No | - | Alive |
| **13** | 3 | Endo,  Endo,  Endo+Epi | T  I  I | No,  Yes,  Yes | 108  107  33 | Yes,  Yes,  Yes | 0  74  1 | Alive |
| **14** | 1 | Endo | I | Yes | 36 | No | - | Alive |
| **15** | 1 | Endo | I | Yes | 36 | No | - | Alive |
| **16** | 1 | Endo+Epi | I | Yes | 38 | Yes | 0 | Alive, progressive RHF |
| **17** | 1 | Endo | I | Yes | 44 | No | - | Alive, progressive RHF |
| **18** | 1 | Endo | I | Yes | 48 | No | - | Alive |
| **19** | 2 | Endo,  Endo+Epi | I  I | Yes,  Yes | 50  48 | Yes,  No | 0,  - | Alive, 2^nd^ RFCA was done in another center |
| **20** | 1 | Endo+Epi | I | Yes | 55 | Yes | 7 | Alive |
| **21** | 3 | Endo,  Endo,  Endo+Epi | I  I  I | Yes,  No,  Yes | 110  87  58 | Yes,  Yes,  No | 0,  3,  - | Alive |
| **22** | 1 | Endo+Epi | I | Yes | 58 | No | - | Alive |
| **23** | 2 | Endo,  Endo+Epi | I  I | No,  Yes | 109  63 | Yes,  No | 5,  - | Alive |
| **24** | 4 | Endo,  Endo,  Endo,  Endo | T  T  T  T | No,  Yes,  Yes,  Yes | 140  139  137  73 | Yes,  Yes,  Yes,  No | 0,  4,  62,  - | Alive |
| **25** | 2 | Endo,  Endo, | I  I | Yes,  Yes | 106  81 | Yes,  Yes | 1,  1 | Alive |
| **26** | 1 | Endo | I | No | 82 | Yes | 0 | Alive |
| **27** | 1 | Endo | T | No | 134 | Yes | 14 | Alive, progressive RHF |
| **28** | 1 | Endo | I | No | 4 | Yes | 0 | SCD 4 months after RFCA |
| **29** | 2 | Endo,  Endo | I  T | Yes,  Yes | 108  91 | Yes,  No | 0,  - | Alive |
| **30** | 3 | Endo,  Endo,  Endo | T  I  I | No,  Yes,  Yes | 145  134  93 | Yes,  Yes,  No | 0,  1,  - | Alive |
| **31** | 1 | Endo | I | Yes | 77 | Yes | 53 | Died 2 months after heart transplantation |
| **32** | 2 | Endo,  Endo | T  I | No,  No | 108  98 | Yes,  Yes | 2,  2 | Alive |
| **33** | 2 | Endo,  Endo | T  T | Yes,  Yes | 100  95 | Yes,  Yes | 5,  16 | Alive |
| **34** | 1 | Endo | I | No | 102 | Lost | Lost | Alive (by police) |
| **35** | 1 | Endo | I | No | 102 | Yes | 70 | Alive |
| **36** | 1 | Endo | T | No | 1 | Yes | 0 | SCD 1 month after RFCA |
| **37** | 1 | Endo | I | Yes | 104 | No | - | Alive |
| **38** | 2 | Endo,  Endo | T  I | Yes,  Yes | 134  106 | Yes,  Yes | 0,  29 | Alive |
| **39** | 3 | Endo,  Endo,  Endo | T  I  I | No,  Yes,  Yes | 112  109  108 | Yes,  Yes,  No | 0,  0,  - | Alive |
| **40** | 1 | Endo | T | Yes | 110 | No | - | Alive |
| **41** | 1 | Endo | I | Yes | 111 | Yes | 17 | Alive |
| **42** | 1 | Endo | I | Yes | 116 | No | - | Alive |
| **43** | 1 | Endo | T | Yes | 118 | No | - | Alive |
| **44** | 1 | Endo | I | Yes | 103 | Yes | 1 | Alive |
| **45** | 1 | Endo | I | Yes | 96 | Yes | 1 | Alive |
| **46** | 3 | Endo,  Endo,  Endo | T  T  T | No  No  Yes | 150  148  126 | Yes,  Yes,  No | 0,  6,  - | Alive |
| **47** | 1 | Endo | T | No | 109 | Yes | 0 | Alive |
| **48** | 1 | Endo | T | No | 20 | Yes | 0 | Died of multi-organ dysfunction due to SCD 20 months after RFCA |

**RVOT: right ventricular outflow tract; RVIT: right ventricular inflow tract; FW: free wall; IW: inferior wall; RVA: right ventricular apex; LVA: left ventricular apex; SCD: sudden cardiac death; lost: Lost of follow-up; I: irrigated catheter, T: thermo-control catheter; RHF: right heart failure.**
